# Extensive Cutaneous Larva Migrans

**DOI:** 10.4269/ajtmh.18-0101

**Published:** 2018-08

**Authors:** Pascal Del Giudice, Thomas Hubiche, Pierre Marie Roger

**Affiliations:** 1 Infectiologie-Dermatologie, Centre Hospitalier de Fréjus-Saint-Raphaël, Saint Lambert, Fréjus, France;; 2 Infectiologie Hôpital Archet 1, Centre Hospitalier Universitaire de Nice, Nice, France

Cutaneous larva migrans (CLM) is a common dermatosis that is acquired in subtropical areas and is caused by animal nematode larvae, mainly including *Ancylostoma braziliense* and occasionally *Ancylostoma caninum*.^1,2^ Larvae penetrate the skin after contact with infected soil and cause creeping eruptions. Typically, a single or a few tracts are present. We report an unusual case of an extensive infection.

**Figure 1.**
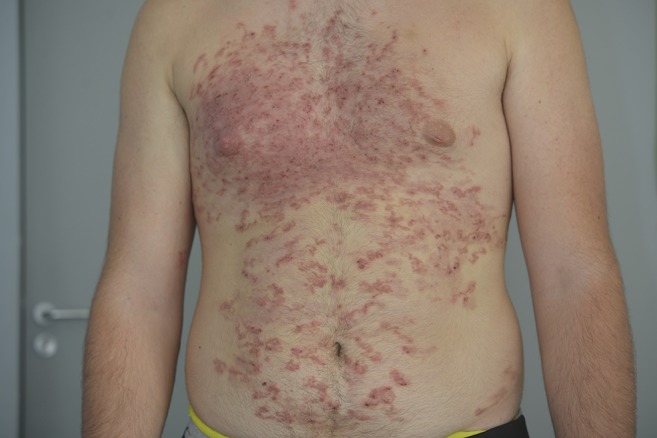
Multiple serpiginous skin tracts. This figure appears in color at www.ajtmh.org.

**Figure 2.**
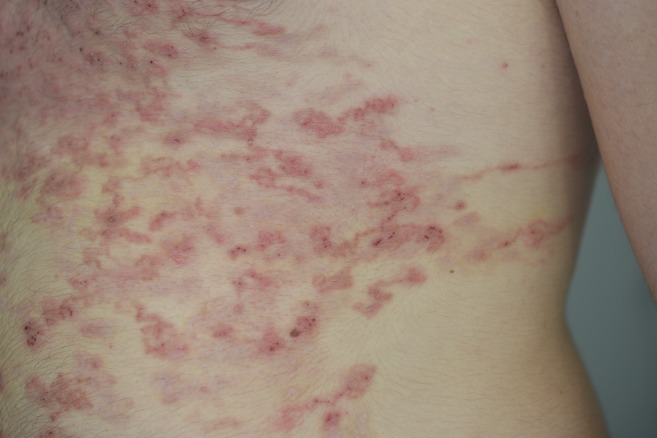
Closer view of the tracts. This figure appears in color at www.ajtmh.org.

A previously healthy 18-year-old man presented with a 1-month history of diffuse and pruritic skin eruption located mainly on the thorax and abdomen. He had returned 1 month earlier from a 2-week trip to Martinique where he had laid on the local beaches. He presented with diffuse linear erythematous and serpiginous tracts (Figures 1 and 2). Laboratory tests revealed leukocytosis of 14.5 × 10^6^/mm^3^ with 47% eosinophils. The clinical diagnosis was typical CLM. He was successfully treated with a single dose of oral ivermectin (200 μg/kg).

In most cases, CLM manifests as a single of a few serpiginous tracts. A more widespread eruption may be associated with a follicular location.^3^ Some parasitoses such as strongyloidiasis or scabies may manifest with a particularly high burden of parasites and are reported in these circumstances such as “hyperinfection.” Similar to these types of parasitoses, the unusual clinical presentation of our patient with widespread lesions suggest a hyperinfection of CLM.
